# Sortilin-Related Receptor Expression in Human Neural Stem Cells Derived from Alzheimer's Disease Patients Carrying the APOE Epsilon 4 Allele

**DOI:** 10.1155/2017/1892612

**Published:** 2017-05-28

**Authors:** Alen Zollo, Zoe Allen, Helle F. Rasmussen, Filomena Iannuzzi, Yichen Shi, Agnete Larsen, Thorsten J. Maier, Carmela Matrone

**Affiliations:** ^1^Department of Biomedicine, University of Aarhus, 6 Bartholins Allé, 8000 Aarhus, Denmark; ^2^Department of Science and Technology, University of Sannio, 82100 Benevento, Italy; ^3^Axol Bioscience, Chesterford Research Park, Little Chesterford Cambridgeshire, Cambridge CB10 1XL, UK; ^4^Department of Anaesthesiology, Intensive Care Medicine and Pain Therapy, Goethe University, Theodor Stern Kai 7, 60590 Frankfurt, Germany

## Abstract

Alzheimer's disease (AD) is the most common form of dementia in the elderly; important risk factors are old age and inheritance of the apolipoprotein E4 (APOE4) allele. Changes in amyloid precursor protein (APP) binding, trafficking, and sorting may be important AD causative factors. Secretase-mediated APP cleavage produces neurotoxic amyloid-beta (A*β*) peptides, which form lethal deposits in the brain. In vivo and in vitro studies have implicated sortilin-related receptor (SORL1) as an important factor in APP trafficking and processing. Recent in vitro evidence has associated the APOE4 allele and alterations in the SORL1 pathway with AD development and progression. Here, we analyzed SORL1 expression in neural stem cells (NSCs) from AD patients carrying null, one, or two copies of the APOE4 allele. We show reduced SORL1 expression only in NSCs of a patient carrying two copies of APOE4 allele with increased A*β*/SORL1 localization along the degenerated neurites. Interestingly, SORL1 binding to APP was largely compromised; this could be almost completely reversed by *γ*-secretase (but not *β*-secretase) inhibitor treatment. These findings may yield new insights into the complex interplay of SORL1 and AD pathology and point to NSCs as a valuable tool to address unsolved AD-related questions in vitro.

## 1. Introduction

Alzheimer's disease (AD) is a progressive neurodegenerative disease associated with cognitive decline and is the most common form of dementia in the elderly. The mechanism underlying the pathogenesis of AD is not completely understood, and beside a growing number of concerns, the amyloid cascade hypothesis remains the prevailing concept for describing some neurodegenerative processes in AD [1]. Amyloid precursor protein (APP) plays a central role in this hypothesis. Indeed, overwhelming evidence—especially from familial forms of early-onset AD (FAD)—has shown that the *β*- and *γ*-secretase-mediated proteolytic breakdown of APP, which generates neurotoxic amyloid-beta (A*β*) peptides, primarily of 38, 40, or 42 amino acid lengths (A*β*38, A*β*40, and A*β*42) [2]. In particular in FAD, mutations in APP or presenilin (PSEN) genes have been shown to either increase A*β*42 production or to increase the ratio of A*β*42 to A*β*40 peptides [3].

The trafficking of APP between the cell surface and intracellular compartments, such as the trans-Golgi network (TGN) and the endosomes, affects the extent of unwanted proteolytic breakdown of APP. The transmembrane protein SORL1, which is a member of the VPS10 domain receptor family, is a crucial regulator of APP trafficking in neurons [4, 5]. SORL1 binds directly to APP and controls APP trafficking from the early endosomes (EE) back to the TGN or the plasma membrane (PM), thereby preventing *β*- and *γ*-secretase-mediated APP cleavage [6, 7]. Indeed, the loss of APP binding to SORL1 alters SORL1 and APP trafficking, leading to increased extracellular shedding of SORL1 protein and substantial endo-lysosomal defects [8]. In mice, these cellular dysfunctions correlate with progressive premature aging, reduced cognitive and learning performance, and neuronal defects [8–13]. Consistently, in AD patients, excessive SORL1 shedding into the cerebrospinal fluid has been noted, and SORL1 has been suggested to be a prognostic biomarker for AD disease development [14]. In mice, overexpression of SORL1 reduces A*β* production and SORL1 knockout cellular and mouse models show increased A*β* production [4].

The main risk factors in developing AD are age, diabetes, and a genetic risk factor, the apolipoprotein (APOE) E4 allele [15, 16]. The presence of one copy of the E4 allele causes a two- to three-fold increase in the risk of developing AD, whereas E4 homozygosity increases the risk up to 12-fold [17, 18]. The mechanisms responsible for A*β* accumulation and neuronal degeneration in AD patients carrying the APOE4 polymorphism are currently not known. APOE4-positive AD patients have been shown to have a higher level of SORL1 in their cerebrospinal fluid [14]. Several population studies have also supported a role for SORL1 in sporadic, late-onset AD (LOAD) [19], and single nucleotide polymorphisms in the SORL1 gene have been linked to an increased risk of LOAD development [20–24]. Correspondingly, reduced levels of SORL1 have been found in hippocampal and cortical tissue from LOAD brains, whereas expression of SORL1 has been reported to be normal in FAD [25, 26]. Despite a large body of evidence from animal and cellular models of AD and neuronal dysfunction, it remains unclear how defects in SORL1 expression and/or shedding can affect APP trafficking and processing in human AD patients. Similarly, it is still not clear whether and to what extent SORL1 plays a causative role in APOE4-related AD development.

Here, we analyzed SORL1 expression and location in human neural stem cells (NSCs) with or without expression of the APOE4 allele. We also analyzed whether a decrease in SORL1 levels in AD neurons might affect the binding of SORL1 to APP and how this was affected by secretase inhibitors.

## 2. Materials and Methods

### 2.1. Induced Pluripotent Stem Cell- (iPSC-) Derived NSCs from Healthy Individuals and AD Patients

iPSC-derived NSCs were purchased from Axol Bioscience (Cambridge, UK). Information about the donors is readily available online (https://www.axolbio.com/).

We used iPSC-derived NSCs obtained from six different donors: four with a diagnosis of AD, one from a healthy donor, and one cord blood donor (CD34+). The last two were used as negative controls (Table 1). Axol Bioscience performed karyotyping of these cells before and after differentiation, and no marked differences were found. As cultured neurons were maintained for a maximum of five weeks, no notable or relevant changes in the karyotype were likely to occur.

Protocols and details of all reagents used for synchronized cell differentiation and culturing were followed as stated by the manufacturer (Axol Bioscience, Cambridge, UK) and available online (https://www.axolbio.com/). The APOE genotype for all NSCs analyzed is reported in Table 1.

### 2.2. Genomic DNA Extraction

NSCs were isolated using Axol Unlock (ax0044) (Axol Bioscience, Cambridge, UK), and genomic DNA was extracted from these cells using Dneasy Blood & Tissue kit (Qiagen, Hilden, Germany), according to the manufacturer's protocols.

### 2.3. Genomic PCR

Genomic PCR was performed using KOD FX Neo (Toyobo, Osaka, Japan) and the following PCR protocol: Mixtures were denatured at 94°C for 2 min, followed by 30 cycles each consisting of 98°C for 10 sec, 65°C for 30 sec, and 68°C for 30 sec. The following primers were used: Position 112, Primer (S) 5′-GCC TCC CAC TGT GCG A-3′ and Primer (AS) 5′-GGC CGA GCA TGG CCT G-3′, position 158 [27] Primer (S) 5′-TAA GCT TGG CAC GGC TGT CCA AGG A-3′ and primer (AS) 5′-ACA GAA TTC GCC CCG GCC TGG TAC ACT GCC-3′.

### 2.4. Direct Sequencing

The genomic PCR product was purified using the ExoSAP-IT DNA purification kit (Affymetrix, Santa Clara, CA, USA) according to the manufacturer's protocol. Direct sequencing was performed using BigDye Terminator kit (Applied Biosystems, Foster City, CA, USA) and analyzed on an ABI 3130xl sequencer (Applied Biosystems, USA).

### 2.5. Immunoprecipitation

For the immunoprecipitation (IP) reactions, protein samples were added to Dynabeads Protein G (30 *μ*g/100 *μ*L) according to the procedure described by the manufacturer (Invitrogen, Hellerup, DK) and eluted with 0.1 M citrate buffer (pH 2.3, adjusted by adding 2 M Tris-HCl).

For IP analysis, we used mouse anti-SORL1 (ab63336), or rabbit anti-APP (clone Y188, ab32136) (Abcam, Cambridge, UK). Western blotting (WB) analysis of IP reactions was performed using rabbit anti-APP (clone Y188, ab32136) and rabbit anti-SORL1 (ab190684) (Abcam, Cambridge, UK). WB analysis of Apo-E has been performed using rabbit anti-Apo-E (clone EPR19392) (Abcam, Cambridge, UK).

### 2.6. Enzyme-Linked Immunosorbent Assay (ELISA)

NSCs (*n* = 200.000) were cultured on 24-well plates in 0.30 mL medium, and the medium was assayed simultaneously for A*β*40 and A*β*42 by ELISA. ELISA was performed as previously described [28]. Mouse anti-6E10, which recognizes residues 1–17 of A*β* (Millipore, Hellerup, DK), was used as capture antibody. A*β* was quantified using the polyclonal rabbit antibody against A*β*1–40 (AB5074P, Millipore, Hellerup, DK) and A*β*1–42 (AB5078P, Millipore, Hellerup, DK) at a concentration of 0.5 *μ*g/mL and 1 *μ*g/mL, respectively. The values of samples were compared against a standard curve, which was generated from samples of known concentrations of A*β*40 or A*β*42 (0.040 ng/mL to 2.0 ng/mL, resp.), and then expressed as A*β*/total protein (pg/*μ*g). For each sample, the levels of A*β*40, A*β*42, and A*β* total were quantified in triplicate on every ELISA plate. To ensure accuracy, standards (duplicate or triplicates) and blanks were run with each plate. Chemiluminescence was detected using tetramethylbenzidine after stopping the colorimetric reaction with 1 M HCl.

### 2.7. Confocal Microscopy and Colocalization Analysis

Neurons from NSCs were fixed for 20 min in phosphate-buffered saline (PBS) containing 4% formaldehyde, permeabilized with 0.05% Triton (5–10 min, 20°C), and processed for double-labeling with the appropriate antibodies. Secondary antibodies coupled to Alexa Fluor dyes (488 or 594) were obtained from Invitrogen (Hellerup, DK). The nuclei were visualized by staining with DAPI (1 *μ*g/mL; Sigma-Aldrich, Hinnerup, DK). Digital images were obtained with a Zeiss LSM lsm780 confocal system using 63 × oil NA 1.3 objectives (Carl Zeiss, Oberkochen, Germany). The colocalization results were quantified using the Zen software, following the procedures previously reported by La Rosa et al. [29], and Pearson's coefficient (*R* coefficient) was used as colocalization coefficient.

For immunofluorescence analysis, we used mouse anti-A*β* (ab11132) and rabbit anti-APP (clone Y188, ab32136), mouse anti-EEA1 (ab70521), mouse anti-Giantin (ab37266), mouse anti-SORL1 (ab63336), rabbit anti-SORL1 (ab190684) and rabbit anti-MAP-2 (ab32454), rabbit anti-*β* III Tubulin antibody (ab18207) and anti-GAP43 (ab16053), anti-GFAP (ab7260) and NeuN (clone 1B7) from Abcam (Cambridge, UK).

### 2.8. Statistical Analysis

Data were expressed as means ± SEM. We performed statistical analysis using GraphPad Prism (version 5.0c, USA) as indicated in the figure legends. In general, experiments involving two experimental groups and a single, nonrepeated, dependent variable were analyzed with Student's *t*-test. When experiments involved three groups or repeated measurements, data were analyzed using either one- or two-way analysis of variance (ANOVA). Post hoc comparisons were made using Tukey's test, when appropriate.

## 3. Results

### 3.1. The NSCs from AD Patients Show an AD-Like Phenotype after Five Weeks in Culture

NSCs were classified as controls (n.1 and n.6) and AD (n.2–5) cells according to the information provided by the manufacturer, Axol Bioscience (Cambridge, UK), and reported in Table 1.

**Figure 1:**
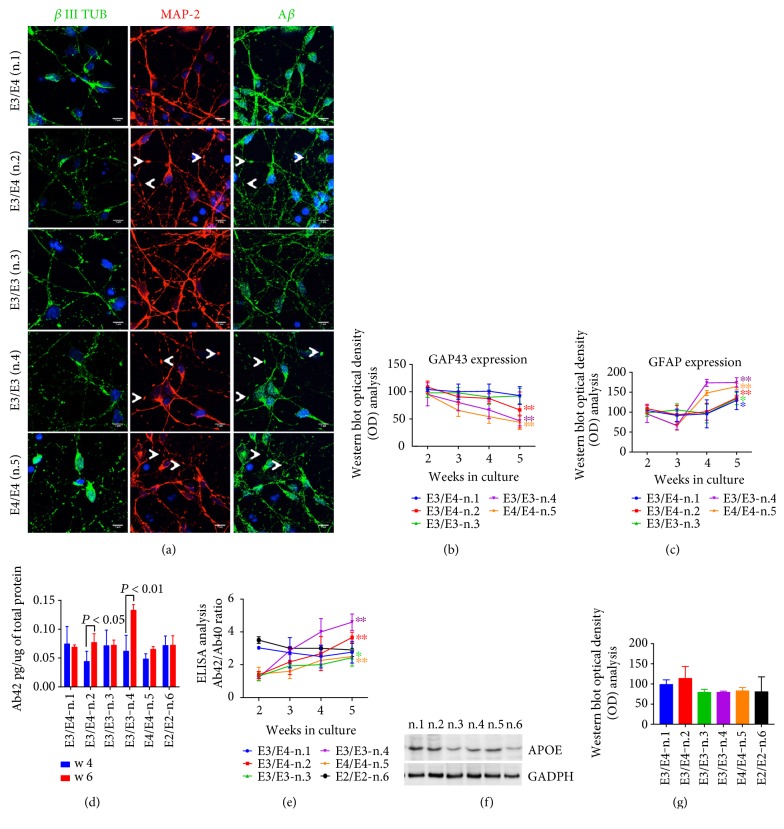
NSCs are completely differentiated after 5 weeks in culture. (a) *β* III Tubulin, MAP-2, and A*β* immunofluorescence analysis in stem cells with or without APOE4 allele expression. Note that the anti-A*β* antibody also detects full length APP. Pictures are representative of five different experiments performed in triplicate. Scale bar: 7 *μ*m. Arrows show the appearance of swollen varicosities along the neurites, likely indicative of a progressive neurodegeneration. (b) Optical density (OD) analysis of GAP43 and (c) GFAP band intensity in NSCs carrying the APOE4 genotype from two to five weeks of culture. Data are normalized to the basal GADPH level and are expressed as % of the corresponding protein level at two weeks in culture. (d) ELISA quantitative analysis of A*β*42 levels in the media of neurons after four and six weeks in culture. (e) The ratio of A*β*42/A*β*40 (Ab42/Ab40) from two to five weeks in culture (2–5). Each data point is the mean ± SEM of triplicate determinations of five independent experiments (*n* = 5). ^∗^*P* < 0.05 and ^∗∗^*P* < 0.01 versus week three of each NSC. One-way ANOVA with post hoc Tukey's test. (f) WB analysis of Apo-E4 protein in culture of NSCs (n.1–6) after five weeks in culture. Densitometry analysis is reported in (g). Data are expressed as % of control (n.1).

The NSCs were plated and cultured for five-six weeks, and the extent to which they differentiated into neurons was assessed via confocal microscopy analysis using anti-*β* III Tubulin (Figure 1(a)) and by WB analysis using the anti-GAP43 antibody (Figure 1(b)), which are markers of neuronal differentiation and maturation, respectively [30, 31]. Immunofluorescence analyses revealed that the vast majority of cells in the outgrowth areas expressed anti-*β* III Tubulin (Figure 1(a)). Interestingly, in the NSCs cultured for five weeks, neuronal degeneration was evident in the n.2, n.4, and n.5, marked by the anti-MAP-2 antibody, as discontinuities in the neurite outgrowth and the appearance of an increasing number of varicosities along the dendrites (Figure 1(a), arrows). Notably, these varicosities contain APP/A*β* structures, as indicated by confocal microscopy analysis using anti-MAP-2 and anti-A*β* antibodies.

To further analyze NSCs' differentiation into neurons, we performed WB analysis for GAP43 and GFAP (a glial proliferation marker) in neurons during the five weeks in culture. As shown in the WB quantification analysis reported in Figures 1(b) and 1(c), after three weeks, there was no difference in the extent of GAP43 and GFAP expression among the cell lines analyzed. However, after four weeks in culture, neuronal GAP43 levels tended to decrease notably in neurons derived from n.2, n.4, and n.5, reaching statistical significance after five weeks (Figure 1(b)). This decrease was paralleled by a progressive increase in GFAP levels (Figure 1(c)). It is also noteworthy that GFAP levels increased in all the neurons analyzed after four weeks in culture (Figure 1(c)). The decrease in the extent of the neuronal marker GAP43 together with the presence of A*β*-positive varicosities along the neurites likely suggested the beginning of neuronal degeneration after five weeks in culture.

To explore whether the NSCs derived from AD patients displayed any disease-related traits, we examined A*β*40 and A*β*42 production during six weeks of culturing by ELISA. A*β*40 and A*β*42 production was below the detection level up to the first three-four weeks of culturing. However, A*β*42 levels were detectable and quantifiable in neurons after four weeks and significantly increased after six weeks in culture in neurons derived from n.2 and n.4 (Figure 1(d)). A*β*40 and A*β*42 were both measurable in control cells as well as in cells from patient n.3 and n.5, after four weeks of culture, without statistically significant changes in the levels during the following two weeks. Of note, Aβ levels were assessed in NSCs from both cord blood cells (n.1) and a healthy donor aged 74 (n.6). Interestingly, the ratio between A*β*42/A*β*40 appeared to increase progressively in all the AD neurons during culture (Figure 1(e)), due to the parallel decrease of A*β*40. In agreement with Israel et al. [32], these results suggest that A*β* secretion occurred only after complete neuronal differentiation and progressively increased only in AD neurons.

Conversely, the lack of significant A*β*42/Α*β*40 increase in AD neurons from patient n.3 suggests that it may require longer culturing for A*β* to reach significant levels in specific cell lines. Relevantly, the A*β*42 increase appeared to be consistent with the neuronal phenotype observed by MAP-2 immunostaining (Figure 1(a)). As either A*β*42 levels or neuronal degeneration became evident after five weeks, we decided to perform the experiments mostly at that time point.

As it has previously been reported that Apo-E is mainly produced by astrocytes [15] in adult human brain, we questioned whether Apo-E was expressed in NSCs from AD and control donors after five weeks in culture. WB analysis clearly indicated the presence of Apo-E protein in all the NSCs analysed (Figures 1(f) and 1(g)).

### 3.2. SORL1 Expression Is Decreased in and Accumulates along the Neurites of Apo-E4-Positive AD Neurons

**Figure 2:**
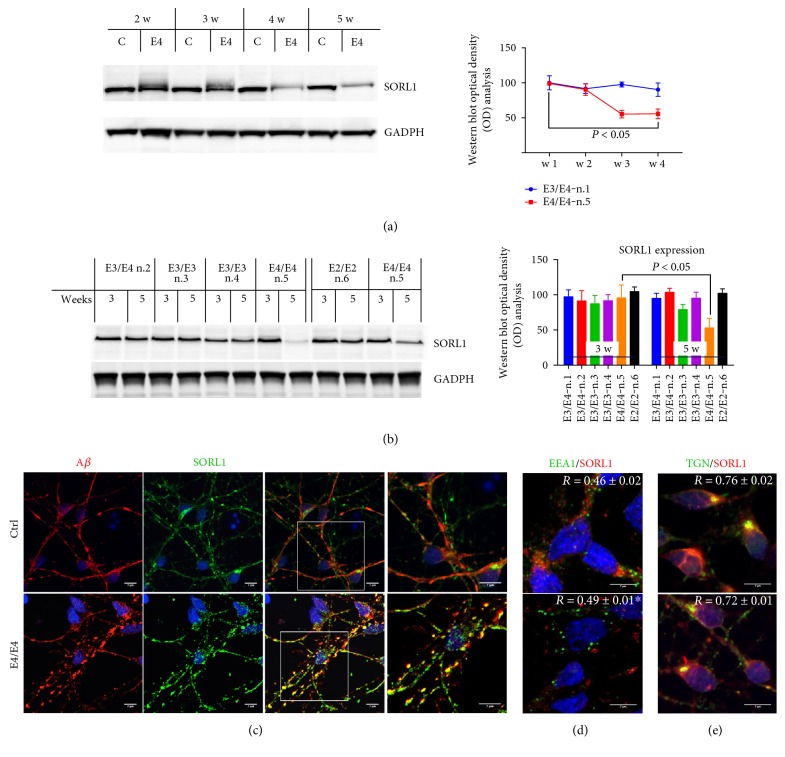
SORL1 expression is decreased in neurons carrying the E4/E4 genotype. (a) WB analysis of SORL1 from control (C, n.1) and E4/E4 (E4) neurons from two to five weeks in culture. SORL1 expression levels were normalized to GADPH and expressed as % of the control (n.1). The quantification of band intensities (OD) is reported on the right. *n* = 4, *P* < 0.05 versus week three. One-way ANOVA with post hoc Tukey's test. (b) WB analysis of SORL1 in neurons with or without APOE4 allele expression after three and five weeks in culture. SORL1 expression levels were normalized to GADPH and expressed as % of the control (n.1). The figure also reports SORL1 expression levels in 74-year healthy donors (n.6). The quantification of band intensities (OD) is reported on the right. *n* = 4; one-way ANOVA with post hoc Tukey's test. *P* < 0.05 versus three weeks. (c) Confocal microscopy analysis of control and E4/E4 neurons after 5 weeks. Neurons are marked with mouse anti-A*β* and rabbit anti-SORL1 antibodies. High magnification is shown on the right. Colocalization analysis of control and E4/E4 neurons by using (d) mouse anti-EEA1 and rabbit anti-SORL1 antibodies and (e) mouse anti-TGN and rabbit anti-SORL1 antibodies. Colocalization analysis was performed using the Zen software. The (*R*) coefficient (Pearson's coefficient) was used for quantitative comparisons between control (Ctrl) and E4/E4 neurons (*R*). The data are expressed as mean ± SEM. *n* = 10 Student's *t*-test ^∗^*P* < 0.05 versus control (n.1).

To evaluate whether SORL1 expression was modified in NSCs from AD patients carrying one, two, or null E4 alleles, during the onset and progression of the AD-like phenotype in vitro, we firstly performed SORL1 WB analysis of neurons from three to five weeks of culturing, using E4/E4 neurons. We noted a progressive decrease in SORL1 levels as culturing proceeded, which was already statistically significant after four weeks (Figure 2(a)). When compared to the other NSCs analyzed, we noted that SORL1 expression was not affected in the control NSCs or in those from the other AD patients (Figure 2(b)).

Interestingly, confocal analysis of SORL1 expression indicated a large increase in SORL1 levels along the neurites in E4/E4 neurons, and in particular, SORL1 appeared to be accumulated inside large varicosities that also contained A*β* (Figure 2(c)), which suggests that both SORL1 and A*β* peptides are linked to the progressive neuronal degeneration of E4/E4 neurons in the patient analyzed. Additionally, this finding might suggest that the reduction in SORL1 expression is probably due to an alteration in its trafficking and localization in differentiated NCSs from the E4/E4 patient. These results seem to be in line with the findings of Caglayan and coworkers [7], indicating that SORL1 can directly bind A*β* and target it to the lysosome for degradation.

The decrease in intracellular SORL1 was further supported by immunofluorescence analysis. We found a decrease in SORL1 staining in the EE-positive vesicles (EEA1) from E4/E4 neurons (Figure 2(d)). In contrast, SORL1 localization in TGN-positive vesicles was not significantly different between E4/E4 and control neurons (Figure 2(e)).

### 3.3. SORL1 Interaction with APP Is Decreased in E4/E4 Neurons

Finally, we questioned whether the decrease in SORL1 levels may affect its binding to full-length APP. We performed coimmunoprecipitation (Co-IP) analysis on control and E4/E4 neurons five weeks after plating, that is, at the time that the levels of SORL1 start to decrease and A*β* is detectable in the media.

**Figure 3:**
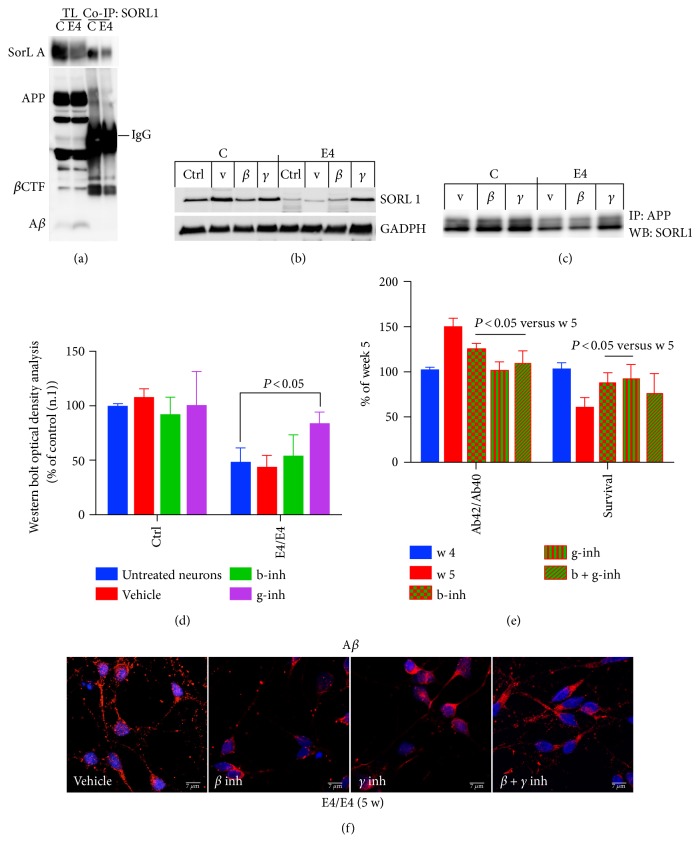
SORL1 binding to APP is decreased in E4/E4 neurons. (a) Protein samples from control (C) and E4/E4 (E4) neurons, after five weeks in culture, were immunoprecipitated with mouse anti-SORL1 antibody (co-IP SORL1) and analyzed with rabbit anti-APP. TL, total lysate; Co-IP, immunoprecipitate. (b) WB analysis of control (C) and E4/E4 (E4) neurons after five weeks in culture (Ctrl, untreated neurons) with vehicle (V, DMSO 0.01%) *β*– (b-inh, 250 *μ*M), and *γ*– (g-inh, 50 nM) secretase inhibitors. (c) The same samples were immunoprecipitated with anti-APP antibody and analyzed with anti-SORL1 by WB. Densitometric analysis (OD) is reported in (d) *n* = 4; one-way ANOVA with post hoc Tukey's test. *P* < 0.05 versus untreated E4/E4 neurons. (e) To counteract A*β*42 production, neurons were exposed to *β*– (b-inh, 250 nM), *γ*– (g-inh, 50 nM), and *β* + *γ* (b + g-inh) secretase inhibitors after four weeks of plating (blue histogram), and A*β*42 levels were assessed one week later, five weeks (red histograms) by ELISA. (f) Confocal microscopy analysis of E4/E4 neurons incubated with or without secretase inhibitors and stained with an anti-A*β* antibody. Nuclei are marked in blue. Note that the anti-A*β* antibody also detects full length APP. Pictures are representative of four experiments (*n* = 4) performed in triplicate. Scale bar: 7 *μ*m. Media with or without inhibitors were refreshed every two days. The level of A*β*42 (expressed as the ratio of A*β*42/40) and the extent of intact NeuN-positive nuclei (survival) are reported in (e). Each data point is the mean ± SEM of triplicate determinations of three independent experiments (*n* = 3) and is expressed as the percentage of values from E4/E4 neurons after 4 weeks of plating.

Precipitation with an anti-SORL1 antibody and analysis with an anti-APP antibody indicated that there was a marked decrease in SORL1 binding to APP in E4/E4 neurons when compared to control neurons (n.1) at five weeks in culture, which was consistent with and partially explained by the decrease in SORL1 expression levels in the total lysates (Figures 2(a) and 3(a)). A significant increase in A*β* was detectable both in the media (expressed as the ratio of A*β*42/A*β*40 levels) and inside neurons carrying the E4/E4 genotype (Ctrl 100 ± 22.3; E4/E4 284 ± 15.8) (Figures 1(e) and 3(a)), supporting previous findings linking the E4 allele to defects in A*β* degradation and clearance [33] and to A*β* cellular uptake through Apo-E receptors [33, 34].

Interestingly, the loss of APP binding to SORL1 was consistent with data previously reported in mice carrying a mutation on the Y_682_ residue on the _682_YENPTY_687_ domain of APP. In both the APP-mutated mice and in E4/E4 neurons, the loss of the APP/SORL1 interaction resulted in SORL1 dislocation. These events are thus the most likely cause of the observed neuronal defects in mutated mice [8].

We also investigated whether the A*β* reduction mediated by *β*- and *γ*-secretase inhibitors can affect SORL1 binding to APP. We firstly assessed the total SORL1 levels in E4/E4 neurons treated with *β*- and *γ*-secretase inhibitors and in the corresponding controls (Figure 3(b)). We noted that *β*- and *γ*-secretase inhibitors did not significantly affect SORL1 expression in control neurons (n.1). However, *γ*-secretase (but not *β*-secretase) inhibitors induced a clear increase in SORL1 expression in E4/E4 neurons. Consistent with the concept that a decrease in SORL1 levels precedes its loss of binding to APP, treatment with *γ*-secretase inhibitor rescued SORL1 binding to APP (Figure 3(c)). As a control, we assessed the ratio of A*β*42/Α*β*40 in the media of E4/E4 neurons before and after the treatment with *β*- and *γ*-secretase inhibitors and noted a marked decrease of A*β* levels in both cases. Consistently, E4/E4 neurons appeared to be protected under *β*- and *γ*-secretase exposure (Figure 3(d)). The decrease in the A*β* extent was also detectable by confocal microscopy analysis (Figure 3(e)).

## 4. Discussion

The influence of SORL1 in AD has been extensively studied in the past, for the most part, by using in vitro cell culture and transgenic or knockout animal models [4, 5, 35]. The main conclusion of these studies was that SORL1 controls APP trafficking and processing in neurons by both preventing accumulation of A*β* [4] and by accelerating targeting of A*β* to lysosomes for degradation [7]. This implicates SORL1 as a potentially important player in the prevention and control of AD.

However, it remains unknown how and whether the previously reported decrease in SORL1 levels in AD patients correlates with the increase in A*β* and reflects the neuronal defects that have been previously described in AD postmortem specimens. Additionally, it is still unclear to which extent SORL1 is implicated in the development of AD in patients carrying the APOE4 allele(s). In this regard, recent reports have suggested that the ApoE-isoform-dependent differences in modulating the cellular A*β* uptake may be related to SORL1 expression and activity [36].

We here studied SORL1 expression in neurons differentiated from NSCs of four patients diagnosed with AD (n.2–5), in one 74 aged healthy donor (n.6) and in neurons derived from cord blood sample (n.1). In vitro, most of the NSCs obtained from AD patients showed a progressive time-dependent increase in the ratio A*β*42/Α*β*40 and neuronal death, making these cells a valuable tool for investigating the molecular mechanism underlying the onset and development of AD. These conclusions appear to be relevant as many studies point toward the clinical utility of NSCs for analyzing the development of the pathology in vitro and for testing drugs or new compounds with the potential to be translated to clinical reality.

In this regard, we found that only one patient, who was homozygous for the APOE4/E4 genotype, showed a decrease in SORL1 levels and altered APP processing, indicating that a single APOE4 allele, alone, is not sufficient to influence the SORL1 pathway. Notably, the accumulation and deposition of A*β* observed in NSCs from two patients diagnosed with AD carrying one or null copy of the E4 allele did not influence the SORL1 expression, suggesting that other factors, currently unclear, may be responsible for the changes in SORL1 expression and activity previously described in AD. Additionally, the decrease in SORL1 levels compromised the APP binding to SORL1 in the E4/E4 neurons, and both SORL1 and A*β* appeared to be largely localized along the neurites.

An interesting aspect emerging from these data is that both *β*- and *γ*-secretase inhibitors were able to counteract A*β* production and to prevent neuronal death in E4/E4 neurons suggesting that both events are dependent on A*β* toxicity. However, only *γ*-secretase inhibitor was able to rescue the APP binding to SORL1 and to restore SORL1 expression levels. This may indicate that the *γ*-secretase pathway is altered in these E4/E4 neurons, as inhibition of *γ*-secretase activity reduces A*β* production, prevents the decrease in SORL1 protein levels, and partially restores SORL1 binding to APP. Interestingly, previous studies reported that SORL1 is a substrate for PSEN 1 and 2 [27, 37]. The finding that *γ*-secretase inhibitor partially restores SORL1 binding to APP suggests that *γ*-secretase activity may be related to SORL1 dislocation and alterations in its binding to APP.

Although it is unclear why a *β*-secretase inhibitor does not affect the SORL1 pathway, the finding that treatment with a *β*-secretase inhibitor protects neurons from death and reduces A*β* levels without affecting the SORL1 pathway suggests that the defects in APP/SORL1 interaction and in SORL1 expression may lie upstream of A*β* production, as previously suggested by Dodson et al. [25, 38].

Interestingly, the decrease in SORL1 expression levels and the consequent loss of binding to APP occurred only in one patient diagnosed with AD (n.5). Conversely, patients carrying one E4 allele did not show alterations in SORL1 expression. Similarly, mutations in PSEN 1 (n.2 and n.4) and PSEN 2 (n.3) genes did not influence SORL1 expression other than the appearance of AD features after five weeks of culture.

## 5. Conclusions

In the light of this complex scenario and beside we are aware that more patients are necessary to draw any conclusion, our results suggest that changes in SORL1 expression are not directly dependent on the APOE4 genetic background; rather, they appear to be related to other factors, most likely specific to each AD patient, which therefore needs to be analyzed individually. Additionally, if these findings could be extended to a larger number of patients carrying the E4/E4 genotype (or not), it may yield new insights into the role played by SORL1 in AD.

Overall, these results emphasize the complexity of the SORL1 pathway in human AD patients and suggest that several factors may contribute to generate the neurodegenerative phenotype reported in NSCs obtained from AD patients.

## Figures and Tables

**Table 1:** Genetic characteristics of the human progenitor stem cells used in our experiments.

Disease	Donor#	Age	Gender	APOE	AXOL line	112	158
Control	n.1	Newborn	M	E3/E4	ax0015	C/T	C/C
Alzheimer's disease	n.2	31	F	E3/E4	ax0114	C/T	C/C
					Presenilin 1 A264E		
	n.3	81	F	E3/E3	ax0115	T/T	C/C
					Presenilin 2 N141I		
	n.4	38	F	E3/E3	ax0112	T/T	C/C
					Presenilin 1 L286V		
	n.5	87	F	E4/E4	ax0111	C/C	C/C
Control	n.6	74	M	E2/E2	ax0018	T/T	T/T

More information can be found at https://www.axolbio.com/shop/category/disease-alzheimers-12.
